# Supplementary material for: Patterns of Intron Gain and Loss in Fungi
Source: PLoS Biol. 2004 Nov 30;2(12):e422. doi: 10.1371/journal.pbio.0020422 (PMC532390; doi:10.1371/journal.pbio.0020422)
Supplement: Table S1 — Also available at http://genes.mit.edu/NielsenEtAl/. (4.3 MB ZIP). [file pbio.0020422.st001.zip › NielsenEtAl/html/1200.html]

AN5979.1.NCU07014.1.MG04114.1.FG05640.1


```
 CLUSTAL W (1.82) Multiple Sequence Alignments - Introns Inserted


Sequence 1: NCU07014.1	146 aa
Sequence 2: MG04114.1	148 aa
Sequence 3: FG05640.1	155 aa
Sequence 4: AN5979.1	139 aa
Alignment Length: 157 aa
Number Identitical Residues: 111 aa
Alignment Score (without introns) 4418


MG04114.1 	MGRVRTK~TVKKSAKVIIERYY~PRLTLDFETNKRICDEIAIIASKRLRNK0IAGYTTHL
NCU07014.1	MGRVRTK~TVKKSAKVIIERYY~PKLTLDFETNKRICDEIAIIASKRLRNK0IAGYTTHL
FG05640.1 	MGRVRTK~TVKKSAKVIIERYY1PRLTLDFETNKRICDEIAIIASKRLRNK~IAGYTTHL
AN5979.1  	MGRVRTK0TVKRSAKVVIERYY~PKLTLDFETNKRVCDEIAIIPSKRLRNK0IAGYTTHL
          	******* ***:****:***** *:**********:*******.******* ********

MG04114.1 	MKRIQRGPVRGISFKLQEEERERKDQFVPEVSALDFSQ-SDSGQLDVDVETKDLLKHLG0
NCU07014.1	MKRIQRGPVRGISFKLQEEERERKDQYVPEVSALDFTQNSESGQLDVDTETKDLLKHLG0
FG05640.1 	MKRIQRGPVRGISFKLQEEERERKDQFVPEVSALDFTQNSESGQLDVDTETKDLLKHLG~
AN5979.1  	MKRIQRGPVRGISFKLQEEERERKDQYVPEISALDVSQ-TESGQLDVDSDTKDLLKSLG0
          	**************************:***:****.:* ::******* :****** ** 

MG04114.1 	FDST-----~---IPVNVIPVTQTQVSERSGRRFGDRPPRRD
NCU07014.1	FDS------~---IPVNVIPVTQAQPVER-GRRFGDRP-RRD
FG05640.1 	VRSPPILTP0FESIPVNVIPVTQQQIPER-GQRYGNRP-RRD
AN5979.1  	FDN------~---LKVNVVAVTQQQ-PER-PRRFRS------
          	. .          : ***:.*** *  **  :*: .
```
